# Hydrocele stone: a rare case report

**DOI:** 10.1093/jscr/rjae098

**Published:** 2024-02-28

**Authors:** Bencherki Youssef, Nedjim Abdelkrim, Tmiri Anas, Moataz Amine, Dakir Mohamed, Debbagh Adil, Aboutaieb Rachid

**Affiliations:** Department of Urology, Ibn Rochd University Hospital, Casablanca 20503, Morocco; Department of Urology, Ibn Rochd University Hospital, Casablanca 20503, Morocco; Department of Urology, Ibn Rochd University Hospital, Casablanca 20503, Morocco; Department of Urology, Ibn Rochd University Hospital, Casablanca 20503, Morocco; Department of Urology, Ibn Rochd University Hospital, Casablanca 20503, Morocco; Department of Urology, Ibn Rochd University Hospital, Casablanca 20503, Morocco; Department of Urology, Ibn Rochd University Hospital, Casablanca 20503, Morocco

**Keywords:** hydrocele stone, hydrocele lithiasis, rare

## Abstract

Hydrocele, characterized by fluid accumulation in the tunica vaginalis, is a common benign scrotal condition. While unusual, hydrocele can lead to rare complications such as infection or lithiasis. A 60-year-old man presented with a 2-month history of left-sided scrotal swelling and discomfort. Physical examination and ultrasound revealed a large, nontransilluminant swelling with hyperechoic images. A provisional diagnosis of scrotal hydrocele with secondary lithiasis was made, and surgical exploration was performed. Intraoperatively, a fluid collection with small, hard stones was found. Cholesterol crystals were identified in the stone. Scrotal lithiasis in hydrocele is rare and is believed to result from stagnant fluid creating an ideal environment for cholesterol crystal formation. Ultrasound is crucial for diagnosis, revealing hyperechoic stones within the fluid collection. Surgical exploration, aspiration of fluid, and stone removal are standard treatments, usually conducted through a small scrotal incision, with a high success rate.

## Introduction

Hydrocele is a common benign scrotal condition that is characterized by the accumulation of fluid in the tunica vaginalis. Although relatively uncommon, secondary complications of hydrocele, such as infection or lithiasis, may occur. Lithiasis in a hydrocele is an extremely rare phenomenon and only a few cases have been reported in the literature. The first report was published in 1975 by Chatterjee [1]. In this case report, we present a patient with hydrocele and scrotal lithiasis and discuss the diagnosis and management of this unusual entity.

## Case report

**Figure 1:**
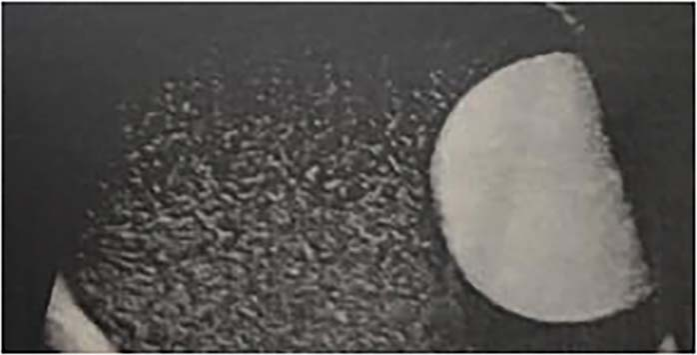
Ultrasound of the hydrocele.

A 60-year-old man presented to the urology clinic with a 2-month history of left-sided scrotal swelling and discomfort. The patient reported that the swelling had gradually increased in size over the past 2 months and was accompanied by occasional pain. Physical examination revealed a large, tender, nontransilluminant swelling in the left hemiscrotum. The right testicle was normal in size and appearance. An ultrasound was performed, which showed a large fluid collection surrounding the left testicle and multiple hyperechoic images within the fluid collection (Fig. 1). A provisional diagnosis of scrotal hydrocele with secondary lithiasis was made, and the patient underwent surgical exploration of the left hemiscrotum.

**Figure 2:**
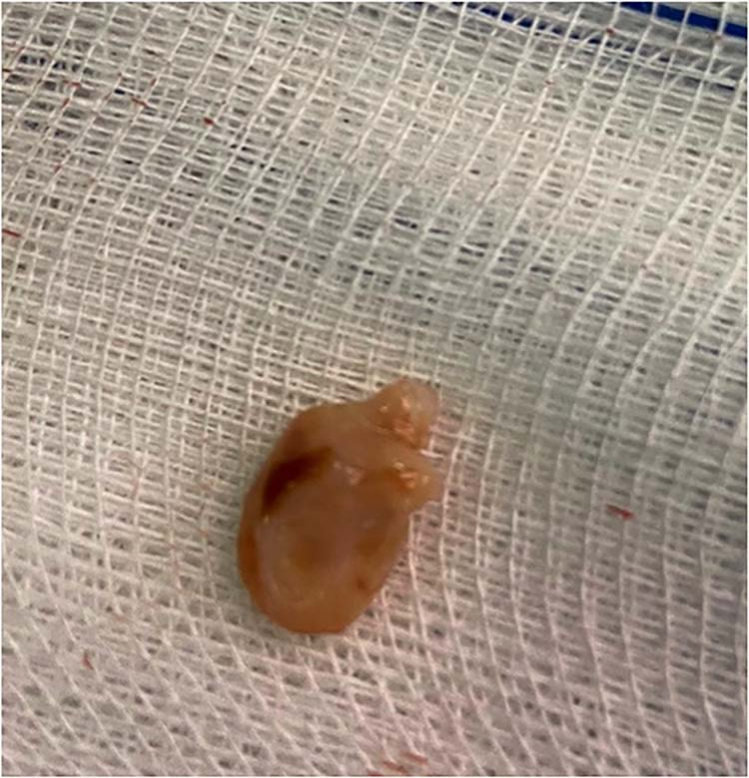
Photograph of hydrocele stone.

Intraoperatively, a large fluid collection was found surrounding the left testicle, which was easily dissected away from the testicle and spermatic cord. The fluid was dark brown in color and contained multiple small, hard stones. The fluid was aspirated and the stone was removed (Fig. 2), and the cavity was then closed. Pathologic examination of the fluid and stone revealed cholesterol crystals, which is consistent with cholesterol lithiasis. The patient had an uneventful postoperative course and was discharged on the third postoperative day. At 6-month follow-up, the patient reported resolution of pain and no recurrence of the scrotal swelling.

## Discussion

Lithiasis in a hydrocele is a rare entity [2], with only a few cases reported in the literature. It is thought to occur due to the presence of a stagnant fluid collection within the tunica vaginalis, which provides an ideal environment for the formation of cholesterol crystals.

These calcifications also are thought to arise from fibrinous deposits in the tunica vaginalis testis following trauma or inflammation, desquamated endothelial cells, or remnants of the appendix testis or appendix epididymidis, which have undergone torsion and become freely movable [3]. The formation of these crystals can result in pain and discomfort, which can lead to the development of a symptomatic hydrocele. The diagnosis of lithiasis in a hydrocele is usually made by ultrasound, which can demonstrate the presence of hyperechoic stones within the fluid collection [4].

Treatment of hydrocele with secondary lithiasis typically involves surgical exploration of the scrotum, with aspiration of the fluid and removal of the stones. This can typically be done through a small incision in the scrotum, and it is typically a low-risk procedure with a high rate of success.

## Conclusion

Lithiasis in a hydrocele is a rare but important entity that should be considered in the differential diagnosis of a symptomatic scrotal swelling. Patients presenting with a scrotal swelling and discomfort should undergo prompt evaluation, including ultrasound, to exclude this diagnosis. Surgical exploration and aspiration of the fluid, with removal of the stones, are the mainstay of treatment for this condition, and this is usually associated with a good outcome.
